# Predictive Factors of Lymph Node Metastasis in Papillary Thyroid Microcarcinoma (PTMC)

**DOI:** 10.3390/medicina61101800

**Published:** 2025-10-06

**Authors:** Odysseas Violetis, Maria Sfakiotaki, Ariadni Spyroglou, Evangelia Pissadaki, Konstantinos Iliakopoulos, Eleni-Konstantina Syntzanaki, Panagiota Konstantakou, Eleni Chouliara, Constantinos Nastos, Nikolaos Dafnios, George Simeakis, Konstantinos Bramis, Despoina Myoteri, George Mastorakos, Paraskevi Xekouki, Krystallenia I. Alexandraki

**Affiliations:** 1 Second Department of Surgery, Medical School, Aretaieio Athens Hospital, National and Kapodistrian University of Athens, 11528 Athens, Greece; odysseas.violetis@gmail.com (O.V.); aspyroglou@yahoo.com (A.S.); kiliakopoulos1@gmail.com (K.I.); panagiotaki05@hotmail.com (P.K.); lenaxou1995@gmail.com (E.C.); dafniosn@gmail.com (N.D.); kbramis@gmail.com (K.B.); mastorakg@gmail.com (G.M.); 2 Endocrinology & Diabetes Clinic, School of Medicine, University Hospital of Heraklion, University of Crete, 71500 Crete, Greece; mariasfak@yahoo.gr (M.S.); esyntzanaki@hotmail.com (E.-K.S.); pxekouki@uoc.gr (P.X.); 3 Third Department of Surgery, Attikon University Hospital, National and Kapodistrian University of Athens, 12462 Athens, Greece; kosnastos@yahoo.gr; 4 Endocrine Department-Thyroid Cancer Outpatient Clinic, 401 General Military Hospital of Athens, 11525 Athens, Greece; gsimeakis@gmail.com; 5 Department of Pathology, Medical School, Aretaieion Hospital, National and Kapodistrian University of Athens, 11528 Athens, Greece; dmyoteri@gmail.com

**Keywords:** papillary thyroid microcarcinoma, lymph node metastasis, pathology, risk factor

## Abstract

*Background and Objectives*: The incidence of papillary thyroid microcarcinoma (PTMC) has increased. Some patients present with lymph node metastases (LNM), while risk factors remain unclear. This study aims to examine clinicopathological markers predictive of LNM in PTMC. *Materials and Methods:* We retrospectively analyzed 170 patients with a histological diagnosis of PTMC. The patients were grouped based on the presence of LNM. *Results:* Our cohort consisted of 133 females and 37 males, aged 47.14 ± 12.81 years. Twenty-seven (15.9%) individuals had LNM. Median tumor size was 5 mm (4.25, 0.15–10), and multifocality was present in 37.1% of patients. Thyroid capsular invasion (TCI) was observed in 22.9% of patients. Extrathyroidal extension (ETE) and aggressive variants were present in 12.9% and 8.8% of patients, respectively. Forty-four patients had a history of autoimmune thyroid disease. From univariate analysis, age < 55 years (OR: 6.317; *p* = 0.015), TCI (OR: 2.824; *p* = 0.020), and ETE (OR: 2.987; *p* = 0.034) were independent predictors of LNM. Multivariate analysis showed that younger patients are at a significantly increased risk of LNM in PTMC (OR: 6.30910; *p* = 0.016). *Conclusions:* The findings of this study highlight the need for greater attention to PTMC in younger patients with ultrasonographic features of TCI and ETE, as they may require a more thorough evaluation of LNM, strict follow-up, and may benefit from a surgical decision of lymphadenectomy.

## 1. Introduction

The incidence of thyroid cancer (TC) has rapidly increased over the past decades, a fact mainly attributed to the widespread use and easy performance of high-sensitivity ultrasonography and subsequent fine needle aspiration (FNA) of thyroid nodules [1,2]. Papillary thyroid carcinoma (PTC) is the most common histological subtype, comprising more than 90% of cases of TC [3], with the subcentimeter PTC, defined as papillary thyroid microcarcinoma (PTMC), accounting for a significant proportion of the increase in PTC incidence [4]. PTMC is generally characterized by an indolent disease course with excellent prognosis, low recurrence, and negligible mortality rates; therefore, a more conservative therapeutic approach is often recommended [5]. Active surveillance (AS) in PTMC without aggressive features is a feasible alternative to surgery at the time of diagnosis [6]. Several studies propose the implementation of minimally invasive techniques in the management of low-risk PTMC [7]. However, the incidence of lymph node metastasis (LNM) in PTMC can be relatively high, reported to be found in 40% of the cases in some cohorts, even in patients with clinically uninvolved lymph nodes (LNs) (cN0), and LNM status is one of the main predictors of recurrence [8]. Whether prophylactic lymphadenectomy should be performed concurrently with total thyroidectomy in PTMC is a matter of debate, due to well-known complications (notably hypocalcemia and laryngeal nerve damage) [9,10]. Risk stratification of PTMC patients is therefore of paramount significance to guide and plan the therapeutic approach to create a balance between overtreatment and disease recurrence. Several clinicopathological factors, such as age, gender, tumor size, and extrathyroidal extension (ETE), have been identified to increase the risk of LNM associated with PTMC, but with conflicting results [9,11,12].

Considering these limitations in the current literature and the huge number of small thyroid nodules detected in daily clinical practice, we conducted a retrospective analysis of clinicopathological data of 170 PTMC patients from two high-volume Greek hospitals, aiming to identify possible risk factors for nodal disease.

## 2. Materials and Methods

In this retrospective study, we have recruited patients with PTMC followed up in Aretaieion University Hospital of Athens and in General University Hospital of Heraklion from 2020 to 2025 with a past or current medical history of PTMC. Specifically, patients were included if they had the following: (1) papillary thyroid carcinoma with a tumor diameter (or largest size of multiplicity) < 1 cm; (2) surgical treatment: lobectomy or near-total/total thyroidectomy with or without neck lymph node dissection (LND); (3) availability of histopathological samples. We excluded patients with concomitant malignant thyroid tumors other than PTC or synchronous PTC with a diameter > 1 cm. We reviewed the medical records, collecting clinicopathological data such as age, gender, the body mass index (BMI, kg/m^2^), history of autoimmune thyroid disease (AITD), as well as surgery reports, data on subsequent RAI administration, and pathological findings of the resected specimens. In a number of patients, only the pathological data were available. Institutional Review Board’s approval was obtained, along with patients’ consent (reference numbers 960/18 June 2021; 574/13 March 2024).

Total thyroidectomy based on the patient’s culturally preferred management or clinical criteria, such as bilateral nodularity, was the standard surgical procedure performed for the majority of the patients [13]. Accordingly, most of the physicians opted for total thyroidectomies to simplify the follow-up. Patients underwent lymphadenectomy in cases of clinically apparent LNM in preoperative LNs mapping or based on clinical intraoperative suspicion of metastasis. In addition, patients were subjected to prophylactic LND of the central compartment based on the presence of gross ETE seen intraoperatively or suspicion of extracapsular invasion based on preoperative ultrasound. Finally, prophylactic LND was performed in cases where preoperative cytology was suggestive of aggressive subtypes. Patients who displayed pathologically high-risk findings, notably ETE and LNM, received radioactive iodine (RAI) treatment after withdrawal of hormone replacement therapy for at least 4 weeks. A diagnostic RAI Whole-Body Scan (RAI WBS) was performed before the administration of RAI and again after 2 to 10 days. Serum thyroglobulin (Tg) and anti-Tg antibodies were measured postoperatively just before the RAI treatment. Oral therapy with levothyroxine postoperatively was given, aiming to suppress their thyroid-stimulating hormone (TSH) levels below 2.0 mlU/L. The patients were followed up every 3–6 months for the first year and every 6–12 months thereafter. The follow-up visits involved measurement of TSH, Tg, and anti-Tg antibody levels, palpation of the neck, and neck ultrasound.

Patients were divided into two groups based on LNs status, pN0 and pN1, respectively, as reported in pathology reviews. For those patients who did not undergo LN dissection or sampling, US resolution reports were utilised, when available. Since not all patients underwent lymphadenectomy in our study, histopathological confirmation of LNM was not uniformly available, thereby introducing the possibility of verification bias. Therefore, the true incidence of clinically significant LNM could be higher than reported. Nonetheless, our results could reflect the real-world practice, where a more selective approach to lymphadenectomy is progressively adopted in low-risk PTMC. Our analysis included age at the time of surgery (<55 vs. ≥55) according to the 8th AJCC (American Joint Committee on Cancer) TNM staging system [14], gender, BMI, evidence of AITD, and the final pathology results including size (≤5 vs. >5 mm), as this cut-off is more frequently used in the literature [11], as well as multifocality, bilaterality, thyroid capsule invasion (TCI), ETE, and aggressive pathological variant of PTC as recently defined [4]. Tumors were considered multifocal if two or more foci were found in one or both lobes, and in that case, the tumor size was determined as the largest diameter of the primary tumor. In multifocal PTMC, we also examined total tumor diameter (TTD) (TTD > 1 cm and TTD ≤ 1 cm). Tall-cell variant of PTC was defined when columnar cells represented at least 30% of tumor cells [4].

Chi-squared test or Fisher’s exact test was used, as appropriate, for categorical variables comparison. The Kolmogorov–Smirnov test was used to test the distribution of the continuous variables. Student’s *t*-test was used for continuous variables comparison with a normal distribution, whereas the Mann–Whitney U-test was used when the parameters did not have a normal distribution. Data are presented as mean values ± SD when displaying a normal distribution, and as median values with interquartile range (IQR) when displaying a non-normal distribution. Odds ratio (OR) and 95% confidence interval (CI) for binary outcomes in univariate and multivariate logistic regression models were calculated and reported using the presence or absence of LNM as the dependent variable. Factors with a *p*-value ≤ 0.05 in univariate analysis were included in the multivariate analysis because of the small number of events. The Kaplan–Meier method was used to analyze recurrence-free survival, and the log-rank test was used to compare recurrence between groups. A *p*-value < 0.05 was considered statistically significant. SPSS Statistics 29.0 software was used for statistical analysis.

## 3. Results

**Figure 1:**
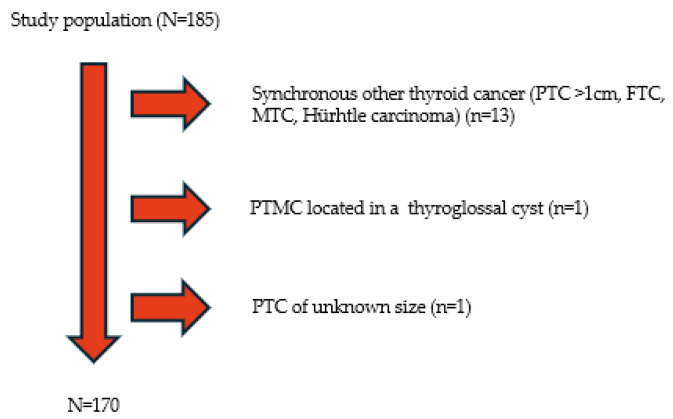
Selection criteria for study patients. PTC: Papillary Thyroid Carcinoma, FTC: Follicular Thyroid Carcinoma, MTC: Medullary Thyroid Carcinoma.

Of the 185 patients in the study population, 170 met the inclusion criteria (Figure 1). Our cohort consisted of 133 females (78%), with a female-to-male ratio of 3.6:1.0. The mean age at diagnosis was 47.14 ± 12.81 years, with an age range of 17–78 years. The mean BMI before surgery was 26.97 ± 4.61 kg/m^2^. All but two patients underwent TT; in two cases, hemithyroidectomy/isthmectomy was performed. Of the 170 patients, 40 (23.5%) had LNs resection documented in the pathology report. LNM occurred in approximately 15.9% (27/170) of the patients. Subsequent RAI treatment was administered in 58.5% of the patients. The most common benign conditions found concomitantly with the microcarcinomas were autoimmune thyroid disease (AITD) (44/106, 41.5%), NIFTP (8/170, 4.8%), follicular adenoma (5/170, 3%), and Graves’ disease (3/170, 1.8%). The median diameter of PTMC (greatest dimension) was 5 mm (IQR, 4.25 and range, 0.15–10 mm). Two-thirds of PTMC were equally distributed in the right (32.7%) and left lobes (35.3%), whereas only 3.5% were located solely in the isthmus. A total of 45 out of 167 patients (26.5%) presented with bilateral involvement. In total, 63 out of 170 (37%) tumors showed multifocality. ETE and TCI were observed in 13% (22/170) and 23.5% (40/170) of the patients, respectively. The most frequent histological type was follicular/classic variant, while tall cell, oncocytic, and sclerosing variants were observed in 10 (5.9%), 3 (1.8%), and 2 (1.2%) patients, respectively. Overall, 15 aggressive subtypes constituted 8.8% of our cohort. The clinicopathological features of PTMCs are summarized in Table 1.

Although most of our patients were women (78.2%), no significant gender differences were documented between PTMC patients with and without LNM (*p* = 0.280). Patients with or without LNM did not differ in most of their baseline characteristics, except for their age at diagnosis (*p* = 0.006) and histological evidence of TCI (*p* = 0.016). Borderline differences were documented for primary tumor size (*p* = 0.079) and ETE (*p* = 0.054). No significant differences were found between groups concerning tumor localization, multifocality, AITD, aggressive histology subtypes, or BMI. A detailed comparison of the demographic and clinicopathological characteristics of patients with positive and negative LNs is summarized in Table 2. In case of multifocality, TTD ≤ 1 cm and TTD > 1 cm were observed in 36 and 27 PTMC cases, respectively. After comparing these two groups, there was no statistically significant difference observed regarding LNM risk (Table 2).

Univariate logistic regression analysis identified age < 55 years (odds ratio (OR): 6.317; 95% CI 1.435–27.817; *p* value = 0.015), TCI (OR 2.824; 95% CI 1.181–6.751; *p* value = 0.020), and ETE (odds ratio (OR): 2.987; 95% CI 1.084–8.228; *p* value = 0.034) as independent factors for LNM in patients with PTMC. The univariate analysis also demonstrated a marginal impact of size > 5 mm on LNM (OR 2.147; 95% CI 0.903–5.103; *p* = 0.084). When multivariate logistic regression analysis was performed to investigate potential factors affecting LNM status, only age < 55 years remained independently associated with LNM (OR: 6.309; 95% CI 1.403–28.372; *p* value = 0.016), whereas TCI only presented an association with LNM (OR: 2.460; 95% CI 0.937–6.459; *p* value = 0.068) (Table 3).

**Figure 2:**
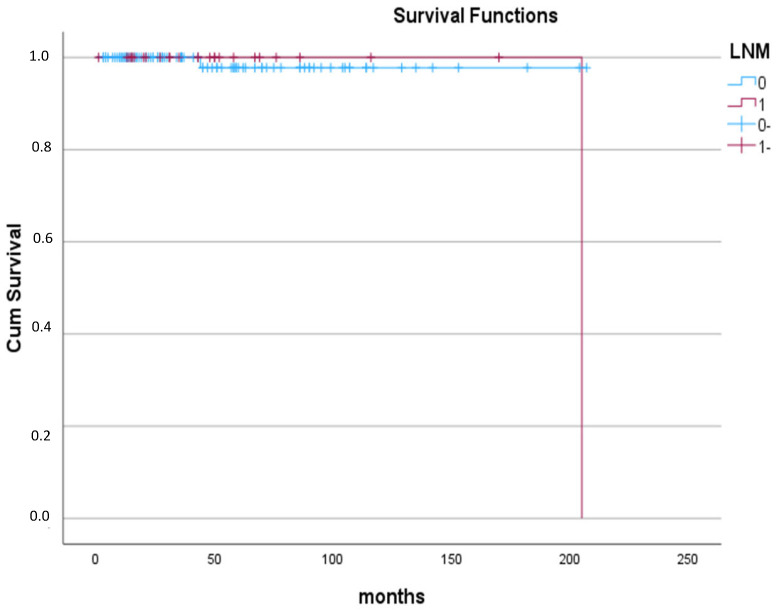
Recurrence-free survival in patients with PTMC based on presence (red line) or absence (blue line) of lymph node metastases. The first patient without lymph node metastasis recurred 44 months after diagnosis, and the second patient with lymph node metastasis, extrathyroidal extension, and thyroid capsule invasion recurred 205 months after diagnosis.

Interestingly, only two patients were diagnosed with recurrence during their follow-up. The first was a female patient with 7 mm encapsulated PTMC who underwent TT with neither TCI nor LNM and without RAI treatment, three years post-thyroidectomy, as revealed by ultrasonography and confirmed by fine needle aspiration (FNA) of suspected LNs. She was reoperated on and subsequently treated with RAI. The second was a male patient diagnosed with bilateral multifocal PTMC with a maximum diameter of 5 mm and TCI and ETE. He underwent total thyroidectomy with lymphadenectomy and RAI treatment. During follow-up after 16 years post-thyroidectomy, Tg levels were successively increasing, and the ultrasound revealed two suspicious LNs. The patient underwent a whole-body radioiodine scan and Positron Emission Tomography/Computed Tomography (PET/CT) scan, which did not detect any abnormalities. FNA and subsequent needle washouts of LNs confirmed metastatic disease originating from the initially resected PTMC. Overall, over a median (IQR, range) follow-up period of 39 (56, 1–207) months, there were no differences between groups in terms of recurrence (with LNM (1%) vs. without LNM (3.8%); *p* = 0.654) (Figure 2).

## 4. Discussion

PTMC incidence is continuously rising, mainly due to the increased use of diagnostic modalities [1,2], displaying an excellent prognosis and a 10-year-survival rate of >95% [4]. However, the 5-year, 10-year, and 20-year probabilities of death were found to be 0.3%, 0.6%, and 1.4%, respectively, in an analysis of 46,662 patients with PTMC from the Surveillance, Epidemiology, and End Results (SEER) program (1983–2015) [15]. Older age at diagnosis, male sex, ETE, and lymph-node involvement were associated with the cumulative incidence of death [15]. Notably, most patients had LN involvement on the initial presentation [16] with rates as high as 65% [17]. Our study showed a rate of LNM of 16% in concordance with some previous reports [18]. In our study, the incidence of clinically significant LNM in PTMC was generally lower than previously reported. This may be attributed to differences in patients’ characteristics, contemporary surgical approaches favoring selective rather than prophylactic lymphadenectomy, earlier detection of PTMC that were not yet metastasized, and the fact that not all patients underwent lymphadenectomy, which could underestimate the true rate of node micrometastases. The most common site of LNM is the central department, but up to 10% of patients with PTMC experience lateral neck LNM [19]. Although the presence of LNM does not seem to affect the overall survival, it is related to a high local recurrence rate and decreased recurrence-free survival [20]. ATA guidelines recommend that lobectomy is sufficient for unifocal, intrathyroidal carcinomas in the absence of prior head and neck radiation, familial thyroid carcinoma, or clinically detectable LNM (ATA 2025) [21]. In our study, all but two patients underwent total thyroidectomy. Some authors are in favor of total thyroidectomy as the initial treatment of PTMC since it is associated with lower recurrence risk, and it facilitates postoperative follow-up using Tg and RAI scans for the possible detection of metastatic foci or recurrence [22]. At present, the indication of prophylactic central neck dissection (pCND) in patients with PTMC with clinically negative LN is still disputed, though it is associated with lower recurrence rates, the possibility of reoperation, and detection of occult LNM [23]. AS has emerged as a viable management strategy for low-risk PTMC (T1aN0M0), when no high-risk features (clinical node and/or distant metastasis and significant ETE) are present [24]. Of course, patients with PTMC under AS ultimately exhibit progression [25,26]. Hence, identification of these aggressive PTMC cases is considered crucial for proper clinical management and improved long-term outcomes. Ultrasonography is used as the primary approach for the preoperative evaluation of central LNM status; however, its low sensitivity (30–50%) renders its diagnostic accuracy unsatisfactory [27].

Unique to PTC, patient age is factored into the staging system. According to the 8th AJCC (American Joint Committee on Cancer) TNM staging system, the break point of age is 55 years [28]. Similarly, we used this specific cut-off in our analysis, showing that age is an independent predictor for nodal disease in PTMC. Our finding that the risk of LNM inversely increased with age was consistent with earlier studies, but a widely accepted age stratification is lacking. Some previous studies have reported similar conclusions using different thresholds, which were 40 [29,30,31], 45 [18,32,33,34], and 55 [16]. Younger patients present the highest rate of LNM, the highest number of metastatic LNs, and a greater proportion of high-risk LNM compared to their senior counterparts [33]. Similarly, other studies have also demonstrated the aggressiveness of PTMC in younger patients, as large-volume LNM is more frequent in those under 40 years of age [29,35]. Other studies have examined age as a continuous variable, showing an inverse relationship with the LNM rate, with the increase in age correlating with a lower LNM risk [36,37]. Recently, a restricted cubic spline (RCS) curve was used to illustrate the relationship between age and LNM in low-risk PTMC. With an inflection point at age 55 years below this value, as age rose, the LNM risk dropped, whereas for those ≥ 55 years, the LNM risk remained stable [38]. The reason why younger patients have a greater predisposition to LNM is yet to be identified; the different gene expression and the higher likelihood of multiple genetic aberrations seem to play a central role [39]. The paradox that younger patients with PTC demonstrate lower mortality rates, despite higher nodal status, warrants further investigation of age-related differences in PTC biology, LN immunologic function, immunological surveillance, and host response [40]. Moreover, several prospective studies have shown that age is a crucial indicator for progression in patients with PTMC under AS, with younger individuals experiencing disease progression [25,26]. Most guidelines recommend using AS for older patients and discourage its use for younger patients, but they do not offer definitive age-specific recommendations. Conversely, some studies failed to identify age as a predictor of LNM in PTMC [41,42,43]. This is of great importance since age may be used as a preoperative decision-making factor influencing the treatment strategy in PTMC patients. In clinical practice, a more thorough evaluation of node disease in younger patients should be undertaken, and the subsequent treatment may differ from that of older patients. Young individuals, for instance, may benefit more from central LND, whereas observation may be more suitable for older patients. Of course, given the retrospective design and small event numbers in our study, larger cohorts or prospective studies are required.

Disparity exists between sexes in TC epidemiology. Women are noted to have nearly four-fold higher rates of clinically detected, early-stage TC than men [44]. This tendency is also reflected in PTMC, as females seem to be diagnosed more frequently [11]. Estrogen receptors may contribute to the development of PTC [33,45]. These receptors are related to tyrosine kinase-signaling pathways, MAPK (mitogen-activated protein kinase) and PI3K (Phosphatidylinositol 3-kinase), which can be upregulated by genes frequently observed in PTC, namely BRAF and RET/PTC. Furthermore, estrogen can stimulate the growth of thyroid cells, promote angiogenesis, and even metastasis [45]. However, the general observation that women are diagnosed more frequently with benign thyroid conditions and are operated on more often may contribute to the observed higher incidence [44]. Despite the female predominance, men tend to have more advanced disease diagnosed at an older age, lower disease-free survival, and higher mortality rate [23]. Previous studies showed that male gender is a significant predictor of LNM [18,19,31,32,33,43,46,47,48] and large-volume LNM (>5 metastatic LNs) [29,35]. The higher male basal metabolic rate potentially leads to hyperactive tumor cell growth and subsequent metastases [32]. Our study did not support a differential impact of gender, consistent with previous studies [34,41,45,49,50].

Tumor diameter has also been correlated with LNM [8,12] and ETE [51]. There is no consensus on the optimal threshold to stratify the risk of LNM, with some studies suggesting 5 mm, while others suggest 6 mm or 7 mm. Nonetheless, a diameter > 5 [42,48,52], >6 [34], or >7 mm [33,53] has been associated with increased risk for LNM (central or lateral). The likelihood of LNM seems to be proportional to the tumor size [26,45]. In contrast, other studies reported no relation between tumor size and LNM [31,49].

ETE is recognized as an indicator of bad prognosis according to ATA [54], and in the 8th edition staging system, PTCs with macro-ETE invading strap muscles or organs are restaged as T3b or T4, while those with micro-ETE are staged as T1/2 (≤4 cm) or T3a (>4 cm) [14]. Since microscopic ETE is difficult to identify prior to surgery, intra-operative frozen sections seem to be essential in PTC, and those with minimal ETE reported in pathology reviews should be closely monitored in case the prophylactic central lymph neck dissection was not performed [52]. PTMCs, along with ETE, are considered highly invasive tumors, and ETE has long been regarded as a marker of clinical progression and recurrence in PTMC [55]. Several studies have indicated that ETE is an independent factor of LNM in PTMC [18,19,20,32,34,35,46,48,52]. A rich lymphatic net is located around the capsule; therefore, the tumor cells that surpass the capsule can easily travel to adjacent LNs [32]. Nonetheless, others failed to support this notion [42]. In our study, ETE had a significant impact on node disease in univariate analysis, and even though this association was not maintained in the multivariate analysis, it underlined its impact on the presence of LNM. We did not distinctly analyze the two entities of ETE (microscopic vs. gross), but studies have shown that both demonstrate similar predictive value concerning LNM prediction [18]. TCI can be examined only after surgery and is related to PTMC aggressiveness [31,47,56], as also documented in our analysis, a finding not consistent with other studies [42,50,53,57]. However, accurate diagnosis is challenging since the thyroid gland lacks a well-defined true capsule and is composed of thin fibrous tissue, which may not be easily observable [47]. Therefore, greater attention should be paid to PTMC placed subcapsularly/peripherally [31]. It may be possible to suspect the probability of TCI with clinically significant LNM preoperatively, as there are some ultrasound features, such as irregular borders or tumors adjacent to the membrane, that have been found to be associated with positive LN status, although definitive diagnosis requires histopathological confirmation [32,33].

Multifocality is defined as the presence of more than two tumor foci in the thyroid gland and is one of the most common features of PTMC [11]. It may arise as independent tumors, from intrathyroidal metastases of a single malignant clone, or multiple independent origins accompanied by intrathyroidal metastasis [52]. In our study, 38% of PTMC were multifocal, like other series [16,18,20,34,48,52,57,58]. ATA guidelines consider PTMC with multifocality but without ETE as low risk [54]; in contrast, multifocality is not considered in the AJCC staging system [14]. However, recent studies have linked multifocality with the aggressiveness of PTMC and the LNM status [18,19,20,32,37,41,43,48,50,52,58] and with a possible impact on prognosis [16,58]. Other studies did not confirm this relationship [31,33,34,35,42,53,57,59]. Additionally, multifocality was found to be an independent factor for non-small volume central LNM (metastatic LNs >5 or ≥2 mm in size), which is linked to a less favorable prognosis, implying that PTMC may ultimately not be low risk [23]. In addition, some studies suggested that TTD is a better indicator of multifocal PTMC aggressiveness, with TTD > 1 cm being correlated with a higher incidence of LNM [60]. Bilaterality is a special situation of multifocality. There is ongoing debate on its role in LNM risk in PTMC [18,33,36,41,48,50,52,56]. Patients with bilateral multifocality compared to unilateral multifocality are more prone to LNM, underlying its importance as a marker of PTMC aggressiveness [58]. Currently, lobectomy is the treatment of choice for papillary microcarcinoma, unless there are clear indications to remove the contralateral lobe, such as bilateral tumor involvement, previous history of head and neck radiation, strong family history of TC, or patient factors that would make follow-up difficult [61]. Our findings that PTMCs are frequently bilateral, in over 25% of the cases, emphasize the need for close clinical and imaging surveillance of the residual thyroid lobe if lobectomy is performed.

Regarding the association of AITD and the risk of LNM in PTMC, current evidence is inconclusive. Several epidemiological studies have confirmed the increased prevalence of AITD in PTC [18,62]. Many studies reported that the presence of AITD in PTMC exerts a protective role because it seems to be a negative predictor of LNM [34,46,47,62]. Patients with AITD had a significantly lower incidence of both central and lateral LNM, fewer LNs, and a lower LN ratio compared to those without AITD [62]. Furthermore, high-risk LNM (high-volume or extranodal extension) was negatively associated with AITD [56]. AITD is characterized by an increased number of lymphocytes and macrophages as well as autoimmune antibodies, which can destroy the follicles, leading to a fibrous cell layer around the tumor nest, thereby limiting its spread, progression, and metastasis [46]. Moreover, Fas-mediated apoptosis induced by AITD may contribute to the overall favorable prognosis in PTC [63]. Other studies showed that AITD was not significantly related to LNM in PTMC [18,31,32,33,41,53,57,64], which is in concordance with our study.

A subset of PTC displays aggressive histology, with distinct molecular and clinicopathological features. Tall cell, diffuse sclerosing, solid, and hobnail variants are known as aggressive subtypes [65]. They tend to metastasize more frequently to LNs and are associated with ETE and worse recurrence-free survival [65,66]. This was confirmed in 11.570 patients with PTMC, where 177 presented aggressive histology. Aggressive subtypes exhibited LNM, ETE, and a possible tendency for structural recurrence post-surgery more often, underpinning the necessity of the final pathology report to decide and plan the extent of surgery [67]. Indeed, FNA is generally inadequate in diagnosing aggressive histology in PTC and, inevitably, pathology confirmation is critical [65]. In our study, we did not find an association between aggressive histology and LNM risk, but identified a non-negligible rate, 8,8%, of patients displaying aggressive features. Similarly, other studies have not confirmed the association between aggressive histology nodal status, ETE, and poorer prognosis in PTMC [16,42,51,57,64].

Lastly, it is advocated that BMI is correlated with PTMC progression, LNM status, and ETE, specifically when ≥25 kg/m^2^, possibly because of the lipotoxicity caused by high triglycerides [55]. In our study, BMI was not found to be linked to LNM status, which is in agreement with other studies [20].

Regarding the limitations, the present study has a retrospective design, which introduces selection bias and limits the generalizability of the findings, as well as the inclusion of a small sample size. Moreover, not all patients underwent LN dissection or sampling; therefore, the real incidence of clinically significant LNM may be underestimated. Also, we did not perform the BRAF analysis in most of the patients. Additionally, we did not perform a further analysis to determine possible differences in the predictors between central LNM and lateral LNM. Finally, because of the design of this study and the limited number of patients, we did not include training and testing sets in the protocol. Despite these limitations, our study provides valuable information as it demonstrates the non-negligible incidence of LNM in PTMC and assesses potential risk factors related to LNM in PTMC presented alongside previously published data.

## 5. Conclusions

In conclusion, our data confirmed that PTMC, despite being regarded as indolent in nature, is accompanied by a high incidence of LNM. The age of the patients represents the most important preoperative predictive factor of nodal disease, with younger individuals being at increased risk. TCI that can also be suspected by ultrasonography preoperatively or early in the postoperative assessment can be an independent predictor of LNM and aid treatment decisions. Finally, ETE also has an impact, even though less than the previous ones. The present study highlights the importance of early risk stratification to select appropriate individualized treatment and follow-up in PTMC patients, ensuring a favorable long-term outcome. As such, creating tools for the early identification of PTMC aggressiveness and LNM can aid in individualizing the clinical management, including the extent of surgery and follow-up.

## Figures and Tables

**Table 1:** Clinical and pathological characteristics of patients.

Variables	Total of Patients (Proportion)
Gender	
Female	133 (78.2%)
Male	37 (21.8%)
Age, years	
Mean ± SD	47.14 ± 12.81
Max-min	17–78
Size	
Median (IQR/range)	5 (4.25/0.15–10)
BMI (Mean ± SD)	26.97 ± 4.61
Position	
Right lobe	54 (32.7%)
Left lobe	60 (35.3%)
Isthmus	6 (3.5%)
Bilateral	45 (27.3%)
Multifocality	
Present	63(37.1%)
Absent	107 (62.9%)
Extrathyroidal extension	
Present	22 (12.9%)
Absent	148(87.1%)
Thyroid Capsule Invasion	
Present	39 (22.9%)
Absent	131 (77%)
Aggressive histology	15 (8.8%)
Tall cell	10 (5.9%)
Sclerosing	2 (1.2%)
Oncocytic	3 (1.8%)
Lymph node metastasis	
Present	27 (15.9%)
Absent	143 (84.1%)
Autoimmune thyroid disease	
Present	44 (41.5%)
Absent	62 (58.5%)
Coexisting diseases	
Follicular adenoma	5 (3%)
NIFPT	8 (4.8%)
Grave’s disease	3 (1.8%)
Surgical Procedure	
Total Thyroidectomy	168 (98.8%)
Other	2 (1.2%)
Lymphadenectomy	
Yes	40 (23.5%)
No	130 (76.5%)
RAI	
Yes	72 (58.5%)

IQR: interquartile range, SD: Standard deviation, BMI: Body Mass Index, NIFTPT: Noninvasive Follicular Thyroid Neoplasm with Papillary-Like Nuclear Features, RAI: Radioactive iodine.

**Table 2:** Demographic and clinicopathological characteristics of patients with and without lymph node metastases (LNM).

Variables	Without LNM	With LNM	Significance
Gender			
Female vs. Male	114 vs. 29	19 vs. 8	0.280
Age at diagnosis (years)			
<55 vs. ≥55	93 vs. 47	25 vs. 2	0.006
Tumor size (mm)			
≤5 vs. >5	73 vs. 68	9 vs. 18	0.079
Position			
Unilateral vs. Bilateral	104 vs. 36	18 vs. 9	0.414
Multifocality	52	11	0.666
TTD of multifocal PTMC			0.507
≤1 cm	31	5	
>1 cm	21	6	
Autoimmune thyroid disease	33	11	0.625
Extrathyroidal extension	15	7	0.054
Thyroid capsule invasion	28	11	0.016
Aggressive histology	14	1	0.470
Tall cell carcinoma	9	1	1.000
BMI	26.77 ± 4.65	27.63 ± 4.54	0.482

BMI: Body Mass Index, TTD: Total Tumor Diameter, *p* < 0.05.

**Table 3:** Univariate and multivariate logistic regression analysis for the factors identified as potentially significant for the development of lymph node metastases in PTMC patients.

	Univariate Logistic Regression Analysis			Multivariate Logistic Regression Analysis		
Variables	Odds Ratio	Significance	95% CI	Odds Ratio	Significance	95% CI
Age at diagnosis < 55	6.317	0.015	1.435–27.817	6.309	0.016	1.403–28.372
Extrathyroidal extension	2.987	0.034	1.084–8.228	2.350	0.128	0.782–7.066
Thyroid capsule invasion	2.824	0.020	1.181–6.751	2.460	0.068	0.937–6.459

Statistical significance *p* < 0.05.

## Data Availability

The original contributions presented in this study are included in the article. Further inquiries can be directed to the corresponding author.
